# Supplementary material for: GPC1 promotes the growth and migration of colorectal cancer cells through regulating the TGF-β1/SMAD2 signaling pathway
Source: PLoS One. 2022 Jun 7;17(6):e0269094. doi: 10.1371/journal.pone.0269094 (PMC9173621; doi:10.1371/journal.pone.0269094)
Supplement: S1 Table — (DOCX) [file pone.0269094.s003.docx]

**S1 Table.** GPC1 is significantly related to M stage in colorectal cancer patients from TCGA.

| **Gene** | **Survival analysis** | **correlation analysis of clinicopathological characteristics** | | | | | |
| --- | --- | --- | --- | --- | --- | --- | --- |
|  |  | Age | Gender | Stage | T | N | M |
| **GPC1** | 0.018 | 0.122 | 0.132 | 0.008 | 0.032 | 0.001 | 0.034 |
| **GPC2** | 0.006 | 0.13 | 0.319 | 0.008 | 0.004 | 0.022 | 0.11 |

Note: Survival analysis was performed by Kaplan–Meier test, and correlation analysis of clinicopathological characteristics was performed by Kolmogorov-Smirnov test; the numbers in the table represent the P value of the correlation analysis.
