# Supplementary material for: GPC1 promotes the growth and migration of colorectal cancer cells through regulating the TGF-β1/SMAD2 signaling pathway
Source: PLoS One. 2022 Jun 7;17(6):e0269094. doi: 10.1371/journal.pone.0269094 (PMC9173621; doi:10.1371/journal.pone.0269094)
Supplement: S2 Table — Note: Survival analysis was performed by Kaplan–Meier test, and correlation analysis of clinicopathological characteristics was performed by Kolmogorov-Smirnov test; the numbers in the table represent the P value of the correlation analysis. (DOCX) [file pone.0269094.s004.docx]

**S2 Table.** Results of 3 independent replicate experiments of cell apoptosis.

| **Cells** | **Times** | **si-Control (%)** | **si-GCP1-1 (%)** | **si-GCP1-2 (%)** |
| --- | --- | --- | --- | --- |
| **SW480** | 1 | 7.82 | 14.41 | 13.65 |
|  | 2 | 8.91 | 10.3 | 12.31 |
|  | 3 | 7.82 | 10.64 | 12.65 |
| **HCT116** | 1 | 4.09 | 7.65 | 7.65 |
|  | 2 | 4.78 | 10.76 | 10.76 |
|  | 3 | 5.33 | 6.04 | 6.04 |
