# Supplementary material for: GPC1 promotes the growth and migration of colorectal cancer cells through regulating the TGF-β1/SMAD2 signaling pathway
Source: PLoS One. 2022 Jun 7;17(6):e0269094. doi: 10.1371/journal.pone.0269094 (PMC9173621; doi:10.1371/journal.pone.0269094)
Supplement: S3 Table — (DOCX) [file pone.0269094.s005.docx]

**S3 Table.** Results of 3 independent replicate experiments of cell cycle.

|  | **SW480** | | | **HCT116** | | |
| --- | --- | --- | --- | --- | --- | --- |
|  | G1 (%) | S (%) | G2/M (%) | G1 (%) | S (%) | G2/M (%) |
| **si-Control** | 72.5 | 16.51 | 10.66 | 62.36 | 11.96 | 25.23 |
|  | 68.25 | 17.64 | 13.62 | 60.55 | 12.93 | 26 |
|  | 64.63 | 20.44 | 14.57 | 58.87 | 13.76 | 26.75 |
| **si-GCP1-1** | 56.54 | 24.73 | 18.01 | 58.2 | 15.86 | 25.07 |
|  | 57.48 | 25.43 | 16.33 | 59.04 | 15.55 | 24.38 |
|  | 57.39 | 25.57 | 17.04 | 55.38 | 18.88 | 25.74 |
| **si-GCP1-2** | 55.54 | 26.45 | 18.01 | 55.2 | 20.73 | 24.07 |
|  | 55.48 | 26.19 | 18.33 | 55.04 | 20.58 | 24.38 |
|  | 55.39 | 26.57 | 18.04 | 55.38 | 19.88 | 24.74 |
