# Supplementary material for: GPC1 promotes the growth and migration of colorectal cancer cells through regulating the TGF-β1/SMAD2 signaling pathway
Source: PLoS One. 2022 Jun 7;17(6):e0269094. doi: 10.1371/journal.pone.0269094 (PMC9173621; doi:10.1371/journal.pone.0269094)
Supplement: S1 File — (DOCX) [file pone.0269094.s006.docx]

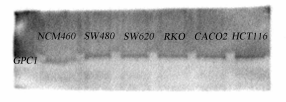

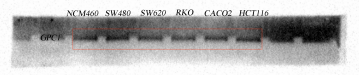

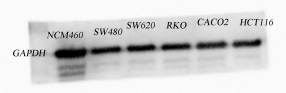

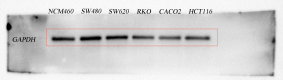


**Fig.2K** Western blotting (Truncated-length of PVDF membranes but including target proteins) was used to analyze GPC1 expression levels in normal mucosal cells and different colorectal cancer cell lines. GPC1 significantly higher expression (P<0.01)in the cell line HCT116 and SW480. Relevant experiment is marked with a red box and lanes have been labeled to match the main text.


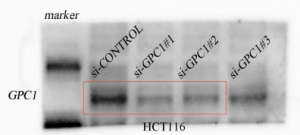

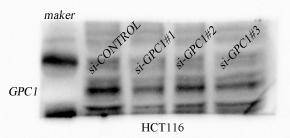

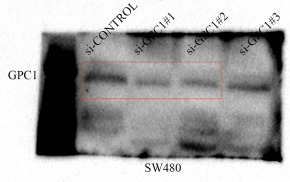

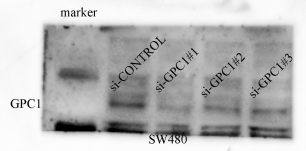


**Fig.3A, 3C** Western blotting (Truncated-length of PVDF membranes but including target proteins) was used to analyze GPC1 expression levels by silencing GPC1 in HCT116 and SW480 cells. GPC1 protein expression decreased significantly by silencing GPC1 in HCT116 and SW480 cells. Relevant experiment is marked with a red box and lanes have been labeled to match the main text.


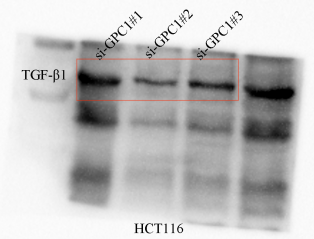

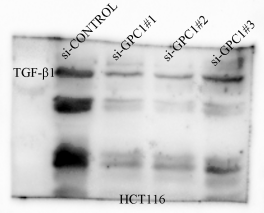

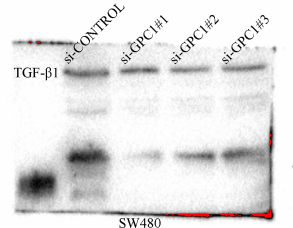

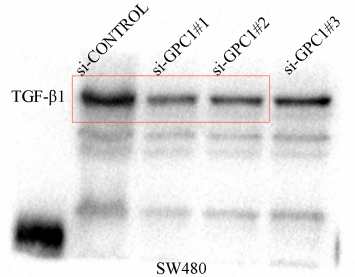


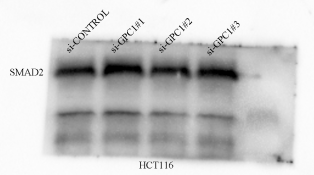

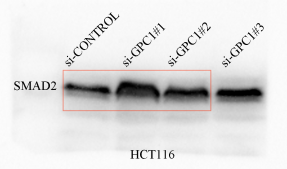

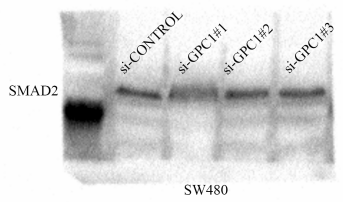

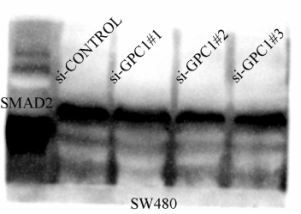


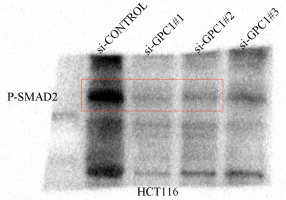

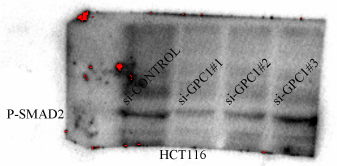

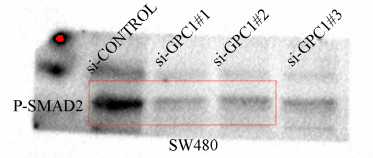

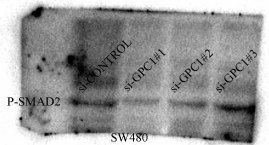


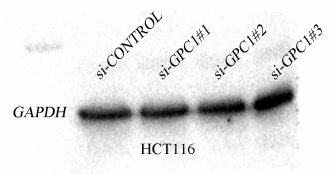

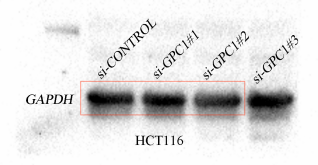

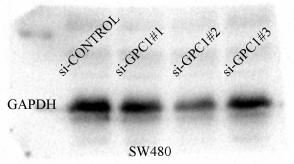

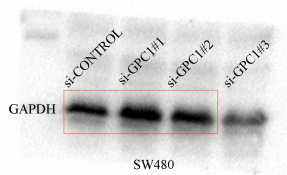


**Fig.6E, 6G** Western blotting (Truncated-length of PVDF membranes but including target proteins) was used to analyze the effect of TGF-β-related proteins. Silencing GPC1 suppressed the expression of TGF-β1 and p-SMAD2 , but increased the expression of SMAD2. Relevant experiment is marked with a red box and lanes have been labeled to match the main text.
